# Clinical features of rheumatoid arthritis-associated interstitial lung disease

**DOI:** 10.1038/srep14897

**Published:** 2015-10-07

**Authors:** Ting Wang, Xing-Ju Zheng, Bin-Miao Liang, Zong-An Liang

**Affiliations:** 1 Department of Respiratory and Critical Care Medicine, West China Hospital, Sichuan University, Chengdu 610041, P. R. China; 2 Department of Radiology, West China Hospital, Sichuan University, Chengdu 610041, P. R. China

## Abstract

Interstitial lung disease (ILD) is the most common extra-articular manifestations of rheumatoid arthritis (RA) in the lung. This study aimed to identify clinical features of RA-associated ILD (RA-ILD). Patients with RA were retrospectively enrolled and sub-classified as RA-ILD or RA without ILD based on high-resolution computed tomography imaging. Pulmonary function testing parameters and levels of RA-related biomarkers, tumour markers, and acute-phase proteins were compared between the two groups. Logistic regression model was used to assess the strength of association between RA-ILD and clinical features of interest. Receiver operating characteristic analysis was performed to assess potential predictive value of clinical features for detecting RA-ILD. Comparison analysis indicated that the percentage of predicted value of total lung capacity, inspiratory capacity, and diffusion capacity of the lung for carbon monoxide (DLCO) were reduced in patients with RA-ILD. Tumour markers CA15–3 and CA125 were increased in patients with RA-ILD. Logistic regression analysis revealed that decreased DLCO was related to the increased likelihood of RA-ILD (OR = 0.94, 95%CI = [0.91, 0.98]). The cut-off point at 52.95 percent of predicted value could sensitively discriminate RA patients with or without ILD. Our study suggested that DLCO value could be a useful tool for detecting ILD in patients with RA.

Interstitial lung disease (ILD) is the most common extra-articular manifestations of rheumatoid arthritis (RA) in the lung, and contributes to the morbidity and mortality in patients with RA^1^. Nevertheless, the estimates of prevalence and incidence of RA-ILD varied immensely because definitions of and measures to diagnose RA-ILD varied in different studies^1^. An early study using the diffusion capacity of the lung for carbon monoxide (DLCO) estimated the incidence of ILD in RA at 41.4%^2^. Recently, more population-based studies have attempted to clarify the definition and incidence of RA-ILD. The cumulative incidence of ILD among patients with RA reported by Bongartz was 7.7% in a population-based cohort with a mean follow up period of 16.4 years^3^. Similarly, the incidence of clinically significant ILD among RA population reported by Olson is near 10%^4^. Poor prognosis has also been observed in patients with RA-ILD. For example, the above two studies both have shown that RA-ILD was associated with shorter survival. Moreover, survival after diagnosis of RA-ILD was only a median of 3 years as reported by Koduri^5^. These results were similar with that reported by Hakala in an earlier study^6^. Given the bad prognosis associated with RA-ILD, earlier recognition was critical.

The majority of patients were diagnosed as RA before the detection of ILD. The clinical complaints of ILD included cough and chronic symptoms of dyspnoea. Impairment of pulmonary function manifested as restrictive pattern with or without low DLCO was also observed in most cases^7^. Although a histological biopsy serves as the gold standard for diagnosis of ILD, many RA patients do not receive a biopsy in clinical practice, especially those with no respiratory symptoms. However, several studies have demonstrated a high level of correlation between high-resolution computed tomography (HRCT) imaging abnormalities and histopathological features^8^,^9^. HRCT imaging manifestations, such as traction bronchiectasis, subpleural fibrosis, honeycombing, and/or ground glass opacification, are often observed in RA-ILD patients and suggestive of usual interstitial pneumonia or nonspecific interstitial pneumonia^1^. Other clinical biomarkers such as rheumatoid factor (RF), anti-cyclic citrullinated peptide (Anti-CCP) have also been researched in RA-ILD patients. High tumour markers were found in RA patients but not related to actual cancer^10^. Ascherman *et al*. suggested a role of tumour biomarkers in RA-ILD^11^. However, the current knowledge of clinical features provides limited pathogenic insight.

Based on these considerations, the current study aimed to investigate the clinical characteristics of patients with RA-ILD, including circulating levels of RF, Anti-CCP, acute-phase protein, tumour markers, and pulmonary function test abnormalities.

## Results

### Characteristics of the Participants

The final cohort consisted of 41 RA participants. Based on evidence of definite interstitial lung abnormalities detected by HRCT imaging scan, 25 patients were identified as having RA-ILD while the other 16 were identified as having RA without ILD. Characteristics of the included patients were presented in Table 1. The average age of the patients with RA without ILD group was 56.19 ± 12.11 and that of RA with ILD group was 63.56 ± 11.90. No significant difference was found between the two groups with regard to age, sex, duration of disease process, or medication. Evaluation of chest HRCT identified 16 reticular abnormalities in RA-ILD patients. In addition, 13 RA-ILD patients had ground glass opacification, 8 had honeycombing, and 1 had traction bronchiectasis.

### Clinical features

Individuals with RA-ILD were more likely to have higher levels of RF, but this association did not reach statistical significance (*P* = 0.10) (Table 1). Likewise, no significant difference was found with regard to Anti-CCP, erythrocyte sedimentation rate (ESR), and C-reactive protein (CRP). Higher levels of carbohydrate antigen 15–3 (CA15-3) and carbohydrate antigen-125 (CA125) were observed in RA-ILD (*P* = 0.01 and 0.03 respectively), levels of carcinoembryonic antigen (CEA) and carbohydrate antigen 19–9 (CA19–9) did not differ significantly (*P* = 0.47 and 0.19 respectively). Comparison of pulmonary function parameters between the two groups indicated that the percent of predicted value of total lung capacity (TLC), inspiratory capacity (IC), and DLCO were reduced in patients with RA-ILD compared with RA without ILD. No significant difference was observed in the percent of the predicted value of vital capacity (VC), forced expiratory volume in the first second (FEV1), and FEV1/forced vital capacity (FVC).

The logistic regression model was made for finding variables of lung function parameters associated with RA-ILD. The forward selection model included preassigned pulmonary function test parameters that reached a significance level of 0.05. This model found that only DLCO (beta –0.06) stepped into the final model and retained statistically significant association with RA-ILD, suggesting decreased DLCO related to the increased likelihood of RA-ILD (OR = 0.94, 95%CI = [0.91, 0.98]) (Table 2).

**Figure 1:**
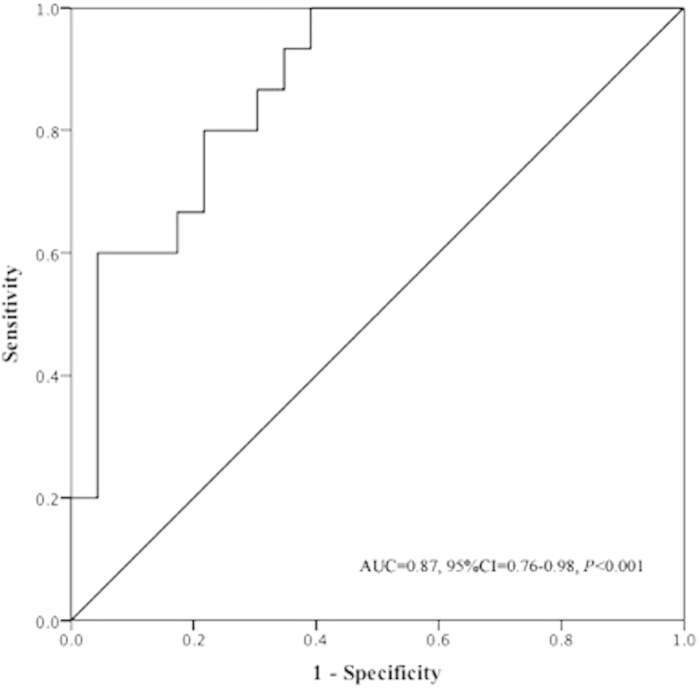
The predictive capacity of levels of DLCO for the presence of RA-ILD.

In order to further demonstrate the predictive potential of DLCO for RA-ILD, a receiver operating characteristic (ROC) assessment was conducted. The generated ROC assessment revealed that the area under the curve value was 0.87 (95%CI = [0.76, 0.98]) (*P* < 0.001), suggesting that levels of the percent of predicted value of DLCO might have moderate capacity for detecting RA-ILD (Fig. 1). In addition, the cut-off point at 52.95 percent of predicted value could sensitively discriminate RA patients with or without ILD (sensitivity was 100%) although the specificity was 60.87%.

## Discussion

Rheumatoid arthritis is a chronic systemic autoimmune disease with a variety of extra-articular manifestations including interstitial lung diseases. Based on comparative analysis, the current study showed that radiographically defined RA-ILD was associated with impaired pulmonary function, reflected as lower percent of predicted value of TLC, IC and DLCO, and higher levels of tumour markers CA15–3 and CA125. Moreover, decreased DLCO value was found to associate with increased likelihood of RA-ILD, and the cut-off point of 52.95 could discriminate RA patients with or without a higher probability of ILD.

Lung is the most common damaged organ in RA patients. Although cardiovascular disease is the primary cause of RA-related deaths, pulmonary disease was found to contribute significantly to a mortality of 10–20% in RA patients^12^,^13^. ILD is a diverse group of chronic lung diseases characterised by progressive fibrosis and is also recognized as early feature of RA^5^. The prevalence of ILD in RA patients varied greatly due to the inconsistency in diagnostic criteria, diagnostic tools and methods of reporting. Clinical features, lung physiology (especially pulmonary function test parameter DLCO), and radiology (especially HRCT imaging scan) are often used to assess whether a RA patient has or does not have ILD. Previous studies using such diagnostic tools reported that the prevalence of ILD varies from 19–44% in RA patients^2^,^3^,^14^. Very often, RA-ILD is overlooked because the presence of clinical respiratory symptoms such as cough and exercise induced dyspnea is unobvious or masked by joint dysfunction. For this reason, although a histological biopsy is considered as the gold standard for diagnosing interstitial pneumonia in RA, it is not a common tool to diagnose ILD in RA patients. Recently, pulmonary function test in combination with HRCT was found to have potentials^15^, which was further confirmed in our study.

Several studies have suggested that genetic factors^16^,^17^,^18^, environmental exposure^19^, and some medicine, such as MTX^16^, implicated in the development of RA-ILD, but the exact mechanisms underlying RA-ILD are still unknown. Turesson *et al*. found that high levels of RF and anti-CCP antibodies increased the risk of ILD development in RA^20^. Ronnelid *et al*. also found that higher levels of anti-CCP antibodies suggested worse clinical disease and the greater progression of extra articular manifestations in RA^21^. However, Bongartz did not report a direct association between anti-CCP with the development of ILD in RA patients^22^. Recently, a study by Harlow suggested that citrullinated protein extracted in RA patient sera might be potential biomarkers for distinguishing RA-ILD from RA without ILD^23^. In our study, although no significant difference in RF and anti-CCP was observed between RA-ILD and RA alone, it is still plausible that immune dysregulation caused by citrullinated proteins might play a role in the development of ILD in RA patients.

Tumour marker is found in the body fluid or body tissues, which can be produced by the tumour or non-tumour cells. Although an elevated level of tumour markers often indicate the presence of cancer, other causes of the elevation were also observed in recent studies. Increased CA19–9, CA125 and CA15–3 were observed in RA patients^24^. Yamamoto has investigated the association between connective tissue disease with interstitial lung disease and tumour maker CA19–9, and found that ILD increased as the expression of CA19–9 increased and that the level of CA19–9 was inversely related with DLCO^25^. Furthermore, after treatment with immunosuppressant, the level of CA19–9 decreased^25^. The current study has found that CA15–3 and CA125 rather than CEA or CA19–9 increased in RA-ILD, indicating that CA15–3 and CA125 might play a more important role in RA-ILD. However, the mechanism underlying its association remains unclear.

In our study, lung function abnormalities (lower TLC, IC, and DLCO) were observed in RA-ILD but not in RA patients without ILD. Moreover, a DLCO value of less than 52.95% had sufficient ability, with sensitivity of 100 and specificity of 60.87, to discriminate RA patients with higher probability of having ILD. These findings were parallel with viewpoints reviewed by Hamblin and Horton, suggesting that a decreased DLCO predicted value could serve as an independent predictor of ILD development in RA^26^. The decreased diffusing capacity might be due to the abnormal ventilation/perfusion ratio, increased diffusion distance, and the destroyed vascular beds. Because diffusing capacity impairment is the most common pathophysiological change in ILD, this finding might supply an adoptive method for detecting ILD manifestations in RA patients. Due to the relatively low-rise specificity, it is wise to combine pulmonary test and HRCT imaging evaluation when subclinical RA-ILD is suspected. On one hand, pulmonary function test, especially the DLCO, appears to be more sensitive in screening RA-ILD because it could detect the pulmonary diffusing function change at the early stage of disease with or without pulmonary structure damage. Similarly, Dawson found that 82% of patients with RA had a reduced DLCO^27^. On the other hand, the HRCT alterations were found to correlate well with the presence of the histopathological subtype and help predict prognosis^26^; therefore, a further HRCT imaging is necessary.

Our study has some limitations though. First, because this was a cross-sectional observational study, we were unable to analyse whether there was casual association between medication usage and the risk of development of RA-ILD. Second, RA severities were not adequately classified. Third, histopathological classification was not performed due to the lack of biopsy specimens. Large, longitudinal studies incorporating more RA patients and general population are required to further elucidate disease pathogenesis and provide additional insight regarding the relationship between RA-ILD and ILD without RA.

In conclusion, alterations of pulmonary function test and HRCT imaging help detect ILD in asymptomatic or symptomatic patients with RA. The DLCO values have demonstrated a good correlation between pulmonary function and HRCT imaging scan. Along with its high sensitivity, the cut-off point at 52.95 percent of predicted value could represent a useful screening tool for detecting RA-ILD. The exact mechanisms underlying RA-ILD should be evaluated in further studies.

## Methods

### Participants

Patients with a confirmed diagnosis of RA were retrospectively queried from the database at West China Hospital of Sichuan University between 2011 and 2014. The inclusion criteria were a diagnosis of RA made by at least two rheumatologists according to the American College of Rheumatology 1987 criteria^28^. Both HRCT imaging scan of the chest and pulmonary function test were performed to assess respective abnormalities suggestive of ILD. Demographics and clinical features were extracted from the database. Patients with neoplasm were excluded. The study protocol was approved by the Institutional Review Board of West China Hospital of Sichuan University, and all participants gave written informed consent. The methods were carried out in accordance with the approved guidelines.

### Pulmonary function test

The pulmonary function test was performed according to American Thoracic Society standards^29^. Each participant was confirmed not using any bronchodilator during an applicable period of time before the test. The percent of predicted values of FEV1, VC, IC, TLC, and DLCO were included in the current analysis. Reference values were derived from predicted value adjusted for Chinese^30^.

### Circulating biomarkers

Serum samples were analyzed by blinded technicians for tumour markers including CEA, CA125, CA19-9 and CA15-3, and acute-phase reactant ESR and CRP.

### Imaging

**Figure 2:**
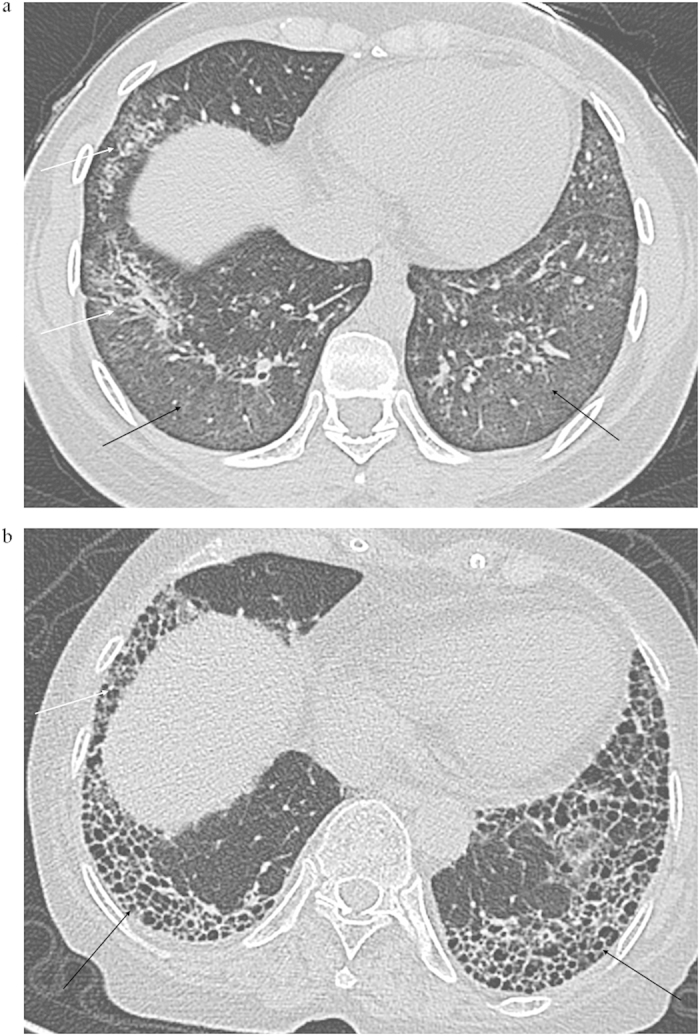
Representative HRCT images for RA-ILD. (**a**) ground-glass opacification (black arrow) and traction bronchiectasis (white arrow), (**b**) Honeycombing (black arrow) and reticular abnormalities (white arrow).

All participants had completed HRCT imaging scan of chest during end inspiration using 1–2 mm-thickness cuts, and those images were evaluated by two blinded radiologists to assess the proximity of ILD manifestations. In brief, having a number of characteristic features, including traction bronchiectasis, reticular abnormalities, and honeycombing, and/or ground glass opacification, was identified as radiographically defined RA-ILD (Fig. 2)^31^. The biomarkers, pulmonary function tests and HRCT imaging scan were planned at admission and performed within a week.

### Statistical analysis

Continuous variables were indicated as mean ± SD for normally distributed data or the median (range) for non-normally distributed data. Categorical variables were presented as frequencies. Comparisons of continuous variables between RA-ILD group and RA without ILD group were conducted by using independent samples *t* test or Mann-Whitney *U* test. The significance of differences in categorical variables between them was analysed using chi-squared test. A logistic regression analysis was performed to assess the strength of association between RA-ILD and clinical features of interest. The predictive capacity of levels of DLCO for the presence of RA-ILD was analysed using ROC curves. Cut-offs with sensitivity and specificity to discriminate RA-ILD from RA without ILD were calculated. All statistical analyses were performed using the SPSS 19.0 software (SPSS Inc., Chicago, IL, USA), and a two-sided *P* value less than 0.05 was considered statistically significant.

## Additional Information

**How to cite this article**: Wang, T. *et al*. Clinical features of rheumatoid arthritis-associated interstitial lung disease. *Sci. Rep*. **5**, 14897; doi: 10.1038/srep14897 (2015).

## Figures and Tables

**Table 1:** Characteristics of included patients of RA with or without ILD.

	RA without ILD (n = 16)	RA with ILD (n = 25)	*P* value
Age, years	56.19 ± 12.11	63.56 ± 11.90	0.06
Sex, male/female	6/10	14/11	0.25
Duration of disease process, months	72 (2, 552)	108 (5, 360)	0.75
**Medication use in hospital**			
Corticosteroid	13	17	0.48
Methotrexate	13	16	0.31
Meloxicam	9	15	0.81
RF	149.00 (20.00, 1680.00)	429.00 (20.00, 4520)	0.10
Anti-CCP	392.90 (7.00, 500.00)	296.35 (1.91, 500.00)	0.87
**Acute-phase proteins**			
ESR	66 (4, 120)	77.5 (34, 120)	0.33
CRP	19.75 (1.22, 134.00)	33.20 (1.91, 261.00)	0.21
**Tumour markers**			
CEA	1.64 (0.71, 3.29)	2.11 (0.57, 7.27)	0.47
CA15-3	11.88 ± 2.72	22.61 ± 13.12	0.01^*^
CA125	23.23 (14.78, 46.68)	54.60 (12.55, 199.80)	0.03^*^
CA19-9	8.92 (0.60, 31.52)	14.04 (0.66, 355.00)	0.19
**Pulmonary function test**			
FEV1, % predicted	89.97 ± 24.73	76.96 ± 22.89	0.09
VC, % predicted	92.21 ± 21.65	80.76 ± 22.24	0.11
FEV1/FVC, %	82.74 (53.61, 95.77)	77.80 (41.67, 95.46)	0.44
TLC, % predicted	100.02 ± 16.25	89.09 ± 15.63	0.04^*^
IC, % predicted	84.78 ± 31.55	64.35 ± 23.55	0.02^*^
DLCO, % predicted	91.71 ± 23.02	52.56 ± 26.52	0.00^*^

^*^P < 0.05. RA, rheumatoid arthritis; ILD, Interstitial lung disease; RF, rheumatoid factor; Anti-CCP, anti-cyclic citrullinated peptide; ESR, erythrocyte sedimentation rate; CRP, C-reactive protein; CEA, carcinoembryonic antigen; CA15–3, carbohydrate antigen 15–3; CA125, carbohydrate antigen-125; CA19–9, carbohydrate antigen 19–9; FEV1, forced expiratory volume in the first second; VC, vital capacity; FVC, forced vital capacity; TLC, total lung capacity; IC, inspiratory capacity; DLCO, diffusion capacity of the lung for carbon monoxide.

**Table 2:** Logistic regression analysis of the association of RA-ILD and pulmonary function test parameters.

Pulmonary function test	OR	95% CI	*P* value
DLCO	0.94	0.90–0.98	0.01^*^
TLC	1.02	0.96–1.09	0.50
IC	0.99	0.96–1.02	0.62

^*^P < 0.05. OR, odds ratio; CI, confidence intervals; DLCO, diffusion capacity of the lung for carbon monoxide; TLC, total lung capacity; IC, inspiratory capacity.

## References

[b1] O’Dwyer D. N. . Rheumatoid Arthritis (RA) associated interstitial lung disease (ILD). Eur. J. Intern. Med. 24, 597–603 (2013). 23916467 10.1016/j.ejim.2013.07.004

[b2] Frank S. T., Weg J. G., Harkleroad L. E. & Fitch R. F. Pulmonary dysfunction in rheumatoid disease. Chest 63, 27–34 (1973). 10.1378/chest.63.1.27 4684107

[b3] Bongartz T. . Incidence and mortality of interstitial lung disease in rheumatoid arthritis: a population-based study. Arthritis Rheum. 62, 1583–1591 (2010). 20155830 10.1002/art.27405 PMC4028137

[b4] Olson A. L. . Rheumatoid arthritis-interstitial lung disease-associated mortality. Am. J. Respir. Crit. Care Med. 183, 372–378 (2011). 20851924 10.1164/rccm.201004-0622OC PMC5450769

[b5] Koduri G. . Interstitial lung disease has a poor prognosis in rheumatoid arthritis: results from an inception cohort. Rheumatology 49, 1483–1489 (2010). 20223814 10.1093/rheumatology/keq035

[b6] Hakala M. Poor prognosis in patients with rheumatoid arthritis hospitalized for interstitial lung fibrosis. Chest 93, 114–118 (1988). 3335140 10.1378/chest.93.1.114

[b7] Lee H. K. . Histopathologic pattern and clinical features of rheumatoid arthritis-associated interstitial lung disease. Chest 127, 2019–2027 (2005). 15947315 10.1378/chest.127.6.2019

[b8] Flaherty K. R. . Radiological versus histological diagnosis in UIP and NSIP: survival implications. Thorax 58, 143–148 (2003). 12554898 10.1136/thorax.58.2.143 PMC1746568

[b9] Hunninghake G. W. . Utility of a lung biopsy for the diagnosis of idiopathic pulmonary fibrosis. Am. J. Respir. Crit. Care Med. 164, 193–196 (2001). 11463586 10.1164/ajrccm.164.2.2101090

[b10] Bergamaschi S. . Tumor markers are elevated in patients with rheumatoid arthritis and do not indicate presence of cancer. Int. J. Rheum. Dis. 15, 179–182 (2012). 22462421 10.1111/j.1756-185X.2011.01671.x

[b11] Ascherman D. P. Interstitial lung disease in rheumatoid arthritis. Curr. Rheumatol. Rep. 12, 363–369 (2010). 20628839 10.1007/s11926-010-0116-z

[b12] Maradit-Kremers H., Nicola P. J., Crowson C. S., Ballman K. V. & Gabriel S. E. Cardiovascular death in rheumatoid arthritis: a population-based study. Arthritis Rheum. 52, 722–732 (2005). 15751097 10.1002/art.20878

[b13] Sihvonen S., Korpela M., Laippala P., Mustonen J. & Pasternack A. Death rates and causes of death in patients with rheumatoid arthritis: a population-based study. Scand. J. Rheumatol. 33, 221–227 (2004). 15370716 10.1080/03009740410005845

[b14] Gabbay E. . Interstitial lung disease in recent onset rheumatoid arthritis. Am. J. Respir. Crit. Care Med. 156, 528–535 (1997). 9279235 10.1164/ajrccm.156.2.9609016

[b15] Leonel D., Lucia C., A. M., Martha-Alicia H. & Blanca M. Pulmonary function test: its correlation with pulmonary high-resolution computed tomography in patients with rheumatoid arthritis. Rheumatol. Int. 32, 2111–2116 (2012). 21499875 10.1007/s00296-011-1933-8

[b16] Nogee L. M. . A mutation in the surfactant protein C gene associated with familial interstitial lung disease. N. Engl. J. Med. 344, 573–579 (2001). 11207353 10.1056/NEJM200102223440805

[b17] Grutters J. C. & du Bois R. M. Genetics of fibrosing lung diseases. Eur. Respir. J. 25, 915–927 (2005). 15863652 10.1183/09031936.05.00133404

[b18] Michalski J. P., McCombs C. C., Scopelitis E., Biundo J. J. Jr. & Medsger T. A. Jr. Alpha 1-antitrypsin phenotypes, including M subtypes, in pulmonary disease associated with rheumatoid arthritis and systemic sclerosis. Arthritis Rheum. 29, 586–591 (1986). 3487321 10.1002/art.1780290502

[b19] Scott J., Johnston I. & Britton J. What causes cryptogenic fibrosing alveolitis? A case-control study of environmental exposure to dust. BMJ 301, 1015–1017 (1990). 2249047 10.1136/bmj.301.6759.1015 PMC1664043

[b20] Turesson C. . Rheumatoid factor and antibodies to cyclic citrullinated peptides are associated with severe extra-articular manifestations in rheumatoid arthritis. Ann. Rheum. Dis. 66, 59–64 (2007). 16901955 10.1136/ard.2006.054445 PMC1798395

[b21] Ronnelid J. . Longitudinal analysis of citrullinated protein/peptide antibodies (anti-CP) during 5 year follow up in early rheumatoid arthritis: anti-CP status predicts worse disease activity and greater radiological progression. Ann. Rheum. Dis. 64, 1744–1749 (2005). 15843452 10.1136/ard.2004.033571 PMC1755292

[b22] Bongartz T. . Citrullination in extra-articular manifestations of rheumatoid arthritis. Rheumatology 46, 70–75 (2007). 16782731 10.1093/rheumatology/kel202

[b23] Harlow L. . Identification of citrullinated hsp90 isoforms as novel autoantigens in rheumatoid arthritis-associated interstitial lung disease. Arthritis Rheum. 65, 869–879 (2013). 23400887 10.1002/art.37881

[b24] Szekanecz E. . Increased production of the soluble tumor-associated antigens CA19-9, CA125, and CA15-3 in rheumatoid arthritis: potential adhesion molecules in synovial inflammation? Ann. NY Acad. Sci. 1108, 359–371 (2007). 17893999 10.1196/annals.1422.037

[b25] Yamamoto S., Kobayashi S., Tanaka M., Akimoto T. & Takasaki Y. [Serum CA 19-9 levels in rheumatic diseases with interstitial pneumonia]. Nihon Rinsho Meneki Gakkai Kaishi 19, 128–135 (1996). 8705689 10.2177/jsci.19.128

[b26] Hamblin M. J. & Horton M. R. Rheumatoid arthritis-associated interstitial lung disease: diagnostic dilemma. Pulm. Med. 2011, 872120 (2011). 21660199 10.1155/2011/872120 PMC3109679

[b27] Dawson J. K., Fewins H. E., Desmond J., Lynch M. P. & Graham D. R. Fibrosing alveolitis in patients with rheumatoid arthritis as assessed by high resolution computed tomography, chest radiography, and pulmonary function tests. Thorax 56, 622–627 (2001). 11462065 10.1136/thorax.56.8.622 PMC1746113

[b28] Arnett F. C. . The American Rheumatism Association 1987 revised criteria for the classification of rheumatoid arthritis. Arthritis and rheumatism 31, 315–324 (1988). 3358796 10.1002/art.1780310302

[b29] Miller M. R. . Standardisation of spirometry. Eur. Respir. J. 26, 319–338 (2005). 16055882 10.1183/09031936.05.00034805

[b30] Zheng J. & Zhong N. Normative values of pulmonary function testing in Chinese adults. Chin. Med. J. 115, 50–54 (2002). 11930658

[b31] Raghu G. . An official ATS/ERS/JRS/ALAT statement: idiopathic pulmonary fibrosis: evidence-based guidelines for diagnosis and management. Am. J. Respir. Crit. Care Med. 183, 788–824 (2011). 21471066 10.1164/rccm.2009-040GL PMC5450933

